# Association Between Random Glucose Level and Leukocytes Count in Female Cancer Patients

**DOI:** 10.7759/cureus.8962

**Published:** 2020-07-02

**Authors:** Mazen M Almehmadi

**Affiliations:** 1 Clinical Laboratory, Taif University, Taif, SAU

**Keywords:** cancer, leukocytes, leukocytosis, breast cancer, uterine cervical cancer, endometrial carcinoma, gastrointestinal cancer, female genital tract cancer

## Abstract

Leukocytosis is common among patients suffering from cancer when leukocytes count exceeds 11,000 cells per mm^3^. This is a usual immune response toward infection and foreign elements. This leads to the release of inflammatory mediators at the targeted site. Studies have found leukocytes count increase in diabetes mellitus patients. Random glucose level indicates the patient is at risk of developing diabetes mellitus. In this study, the association between random glucose level and leukocytes count in female cancer patients is evaluated. About 210 cancer patients included in this study and the results have indicated a positive association between high glucose level and high leukocyte count. This indicates poor prognosis of the patients as high glucose levels increase tumor cell proliferation and high leukocytes count can induce inflammation leading to the progression of cancer and increase mortality rate.

## Introduction

The mortality rate due to cancer is high worldwide. Leukocytes have a major role in immune system response against cancer as they recognize and infiltrate tumors [1]. Expansion of leukocytes in the blood is defined as leukocytosis and upon activation, they well release several inflammatory mediators. Normal level of white blood cells (WBC) is different and age-dependent, the normal level in adults is 4500 to 11000 cells per mm^3^; this level is higher in infant and pregnant females [2,3]. A study has recommended that reference range for leukocytes count should be set according to the laboratory specific analyzer. This is due to different validation processes [2]. Leukemoid reaction is a phenomenon that occurs due to factors such as infection and solid tumor formation, and it is defined as when leukocyte numbers expand to reach 50,000 up to 100,000 cells per mm^3^ [3,4]. The involvement of bone marrow or high levels of a granulocyte-colony-stimulating factor (G-CSF) can induce leukocytes expansion [3,5]. Leukocytosis is detected in solid tumor patients which can lead to severe complications. The expansion of leukocytes can reach levels of leukemoid reaction between 50,000 to 100,000 per mm^3^ which can lead to an inflammatory response [3,5]. Human papillomavirus (HPV) is the main etiological factor of cervical cancer, and experimental models with HPV-positive models have shown high recruitment of leukocytes toward these cell lines [6]. Various studies have detected several abnormalities in hematological patterns in cancer patients. Leukocytosis was detected in patients with solid tumors according to a study that detected anemia and thrombocytosis as well [7]. Leukocytosis was also detected in non-small cell lung carcinoma [8]. Moreover, it was also detected in colorectal cancer patients which also accompanied by dehydration and malnutrition [9]. Another study has reported leukocytosis with anal cancer patients, which can assist in predicting possible relapse after treatment [10]. Leukocytosis was also correlated with increase mortality rate when co-existing with thrombosis [11].

Diabetes mellitus (DM) impact on health is thoroughly studied and documented. Glucose intolerance and chronic complications of DM are associated with an expansion of leukocytes number, which can also advance and cause nephropathy [12,13]. In this study, an investigation about the association between glycemic status and leukocytes number in cancer patients was conducted.

## Materials and methods

Study design

This retrospective study was approved by the directorate of health affairs in Taif city for the period of 2018 to 2019; a consent form was provided. Firm inclusion measures were applied to include the data in the study. Firstly, patients in the study diagnosed with any type of cancer at King Faisal Hospital (KFH) between 2018 and December of 2019; secondly, only female cancer patients were included; thirdly, they had a measurement of both glucose levels and leukocytes count. The number of recruited participants in this research was 210.

Samples analysis

When patients were requested to provide biopsy samples, a minimum of 3 mL of venous blood was collected, analyzed freshly, and not stored through ROCHE COBAS® platform e501. Patients were advised to fast for 10 hours before collecting the blood. This study collected the following information: age of the patient, type of diagnosed cancer, glucose level, and WBC count.

Statistical analysis

Microsoft excel for office was used for sorting of data, calculating frequencies, percentage, standard deviation (σ), chi-square analysis to compare frequencies, and one-way ANOVA was used to compared means. Results when P-value < .05 were considered significant.

## Results

Demographic analysis

The number of participants in this study was 210 female patients from 2018 to December 2019. The youngest participant's age was 13 years and the oldest was 108 years old. All those patients were living in Taif. All study participants were females who were confirmed to develop malignant tumor (Table 1).

**Table 1:** Participants are distributed into three groups according to their age. SD: Standard deviation

	Age groups			Total
	<40	40 - 64	>64	
Number of participants	36 (17.14%)	125 (59.52%)	49 (23.34%)	210
Mean ± SD	33 ± 6	53 ± 6.8	75 ± 9	

Frequencies of cancer types

The type of cancer of this study patients is illustrated in Table 2. Most patients were breast cancer patients and about 80% of the cases were invasive duct carcinoma patients, followed by female genital tract cancer patients, about 63% of them were endometrial carcinoma, followed by 22.22% cervical cancer. Gastrointestinal tract cancer patients were 16.5%, and 28.57% of those were sigmoid cancer patients, and 25.71% diagnosed with rectal mass cancer. Head and neck cancer patients were 9.43%, the highest percentage of patients (65%) were diagnosed with thyroid cancer. All urinary tract patients were diagnosed with bladder cancer. Blood tumor patients were only 3.3% and skin cancer 1.41%.

**Table 2:** Frequency of cancer patients are distributed according to the tissue and diagnoses.

Type	Diagnosis	Number of cases	% by type
Breast cancer (BC)	Invasive duct carcinoma	65	80.24
	Invasive lobular carcinoma	10	12.34
	Invasive micropapillary carcinoma	2	2.46
	Invasive mammary carcinoma	2	2.46
	Mucinous adenocarcinoma	1	1.23
	In situ duct carcinoma (comedocarcinoma)	1	1.23
Female genital tract cancer (FGT)	Endometrial carcinoma	34	62.96
	Cervical cancer	12	22.22
	Uterus with bilateral fallopian tube	3	5.55
	Uterine carcinoma	2	3.70
	Left ovarian cyst	2	3.70
	Anterior vaginal wall	1	1.85
Gastrointestinal tract cancer (GIT)	Sigmoid cancer	10	28.57
	Rectal cancer	9	25.71
	Colon cancer	5	14.28
	Caecum cancer	2	5.71
	Hepatocellular carcinoma	2	5.71
	Adenocarcinoma of rectosigmoid	1	2.85
	Ascending colon cancer	1	2.85
	Duodenal cancer	2	5.71
	Esophageal cancer	1	2.85
	Gastric mucosal cancer	1	2.85
	Hyperplastic polyp	1	2.85
Head and neck cancer (H&N)	Thyroid cancer	13	65
	Tubulovillous adenoma	3	15
	Hurthle cell adenoma	1	5
	Nasopharyngeal cancer	1	5
	Palate cancer	1	5
	Tongue carcinoma	1	5
Urinary tract cancer (UTI)	Bladder cancer	10	100
Blood tumor (BT)	Spindle cell tumor	2	28.57
	Multiple myeloma	1	14.28
	Hodgkin's lymphoma	1	14.28
	Left axillary lymph nodes	1	14.28
	Non-Hodgkin's lymphoma	1	14.28
	Right inguinal lymph node	1	14.28
	Right axillary lymph node	1	14.28
Skin cancer (SC)	Skin, subcutaneous left cheek cancer	1	33.33
	Skin from right gluteal region cancer	1	33.33
	Scalp cancer	1	33.33

Association between random glucose levels (RGL) and leukocyte count

**Figure 1:**
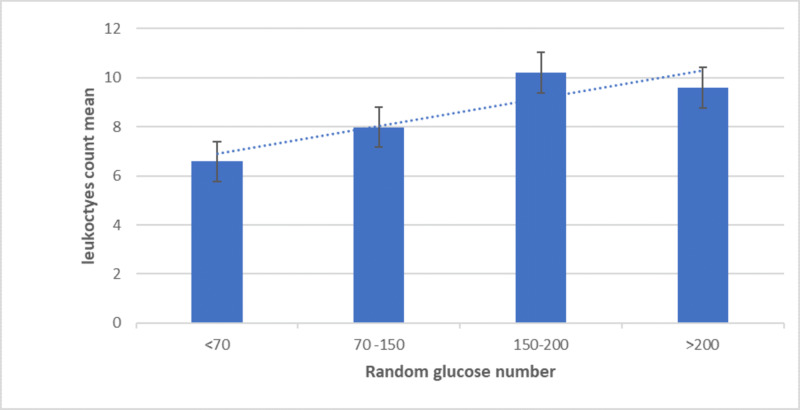
In the bar-graph, the mean of leukocytes count is positively increasing in association with the increase in RGL concentration in the blood. The lowest level is in <70 RGL group (P-value .0208). RGL: Random glucose levels Leukocytes count K/uL (K = 1000) Number of <70 = 12, Number of 70-150 = 154, Number of 150-200 = 22, Number of >200 = 22.

RGL was associated with leukocytes number. The levels of RGL were divided into four groups, and the mean levels of leukocytes number from all patients were calculated (Figure 1). The RGL was divided into <70, 70 to 150, 150 to 200, and >200 and compared by applying one-way ANOVA test. Significantly the highest mean of leukocytes number among all patients’ groups were those with RGL 150 to 200, followed by >200, 70 to 150, and the lowest group with <70 (P-value .0208).

In Table 3, the association between RGL and leukocytes count is illustrated. According to age groups, in <40 years, significantly 80.5% are having normal leukocytes number, 11.12% having leukocytosis, and 8.3% having leukopenia. In 40 to 64 years, 80.62% of the patients are significantly showing normal leukocytes number, followed by 12.4% having leukocytosis and 4% having leukopenia. The hyperglycemic patients (69.23%) in this age group are significantly showing normal levels of leukocytes, 23.07% showing leukocytosis, and 7.7% showing leukopenia. For the age group >64 years, significantly 77.55% patients are showing normal levels of leukocytes, 12.23% showing leukocytosis, and 10.2% showing leukopenia. The hyperglycemic patients (66.66%) in this age group are showing significantly normal levels and 33.33% showing leukocytosis.

**Table 3:** Association between RGL and leukocyte count is identified according to age, cancer type, and menopause status. Leukocytes mean K/uL BC: Breast cancer; FGT: Female genital tract; GIT: Gastrointestinal tract; H&N: Head and neck; UTI: Urinary tract infection; BT: Blood tumor; SC: Skin cancer.

Characteristics			Number of cases	Leukocytes mean ±SD	Leukocytosis (%)	Normal (%)	Leukopenia (%)	P-value
Age groups	<40	All	36	7.95 ± 3.35	4 (11.12)	29 (80.5)	3 (8.3)	0.00001
		Hyperglycemic	3	9.72 ± 6.04	2 (66.66)	0	1 (33.33)	0.36787
		Hypoglycemic	3	8.21 ± 1.09	0	3 (100)	0	0.05178
	40 - 64	All	125	8.35 ± 3.94	16 (12.8)	104 (83.2)	5 (4)	0.00001
		Hyperglycemic	26	9.54 ± 3.54	6 (23.07)	18 (69.23)	2 (7.7)	0.00003
		Hypoglycemic	1	-	0	1 (100)	0	0.36787
	>64	All	49	8.39 ± 4.5	6 (12.24)	38 (77.55)	5 (10.2)	0.00001
		Hyperglycemic	12	12.29 ± 6.69	4 (33.33)	8 (66.66)	0	0.0183
		Hypoglycemic	2	4.67 ± 0.41	0	1 (50)	1 (50)	0.6065
Cancer types	BC	All	81	7.65 ± 3.38	4 (5)	69 (86.25)	7 (8.75)	0.00001
		Hyperglycemic	12	8.03 ± 4.22	1 (8.33)	9 (75)	2 (16.66)	0.00865
		Hypoglycemic	2	4.2 ± 0.12	0	2 (100)	0	0.1353
	FGT	All	54	8.91 ± 2.72	11 (20.37)	41 (75.9)	2 (3.7)	0.00001
		Hyperglycemic	9	10.5 ± 2.63	2 (22.22)	7 (77.77)	0	0.0131
		Hypoglycemic	1	0	0	1 (100)	0	0.3678
	GIT	All	35	9.5 ± 5.7	4 (11.42)	27 (77.14)	4 (11.42)	0.00027
		Hyperglycemic	9	10.82 ± 5.26	1 (11.11)	8 (88.88)	0	0.0017
		Hypoglycemic	1	0	0	0	1	-
	H&N	All	20	6.57 ± 2.18	1 (5)	17 (85)	2 (10)	0.00006
		Hyperglycemic	1	0	0	0	1	-
		Hypoglycemic	1	0	0	1 (100)	0	-
	UTI	All	10	7.97 ± 1.99	0	9 (90)	1 (10)	0.00067
		Hyperglycemic	0	0	0	0	0	-
		Hypoglycemic	0	0	0	0	0	-
	BT	All	8	7.58 ± 3.69	2 (25)	5 (62.5)	1 (12.5)	0.19691
		Hyperglycemic	2	8.9 ± 5.12	1 (50)	1 (50)	0	-
		Hypoglycemic	0	0	0	0	0	-
	SC	All	3	7.42 ± 2.17	0	3 (100)	0	-
		Hyperglycemic	1	0	0	1 (100)	0	-
		Hypoglycemic	0	0	0	0	0	-
Menopause	Pre	All	81	8.09 ± 3.9	9 (10.7)	67 (79.76)	9 (10.71)	0.00001
		Hyperglycemic	4	8.19 ± 3.76	2 (50)	0	2 (50)	0.3678
		Hypoglycemic	4	7.745 ± 1.28	0	4 (100)	0	0.01831
	Post	All	130	8.43 ± 3.99	17 (43.34)	104 (80)	9 (6.92)	0.000001
		Hyperglycemic	33	10.3 ± 4.99	10 (30.3)	22 (66.66)	1 (3.03)	0.000041
		Hypoglycemic	2	4.66 ± 0.43	0	1 (50)	1 (50)	0.60653

Evaluation of the association according to the cancer type has shown the following: in breast cancer, the highest group has shown normal leukocytes number in all patients and hyperglycemic patients, followed by leukopenia in all patients group and hyperglycemic patients group. In female genital tract cancer patients, 75.9% patients showed normal leukocytes count and 20.37% showed leukocytosis. In the hyperglycemic group, 77.77% showed normal leukocytes count and 22.22% showed leukocytosis. In gastrointestinal tract cancer patients, significantly 77.14% patients showed normal leukocytes count and 88.88% hyperglycemic patients showed normal leukocytes count. In the head and neck group, significantly 85% patients showed normal leukocytes count. In the urinary tract patients, significantly 90% patients showed normal leukocytes count. No leukocytosis was detected in all hypoglycemic patients.

Furthermore, menopause status was associated according to RGL and leukocytes count. Significantly, 79.76% premenopausal cancer patients showed normal leukocytes number, and the same percentage of the patients showed leukocytosis and leukopenia. In post-menopausal cancer patients, 80% showed normal leukocytes number, followed by 13% with leukocytosis and 7% with leukopenia. Hyperglycemic patients were 66.66% with normal leukocytes number and 30.3% with leukocytosis.

**Figure 2:**
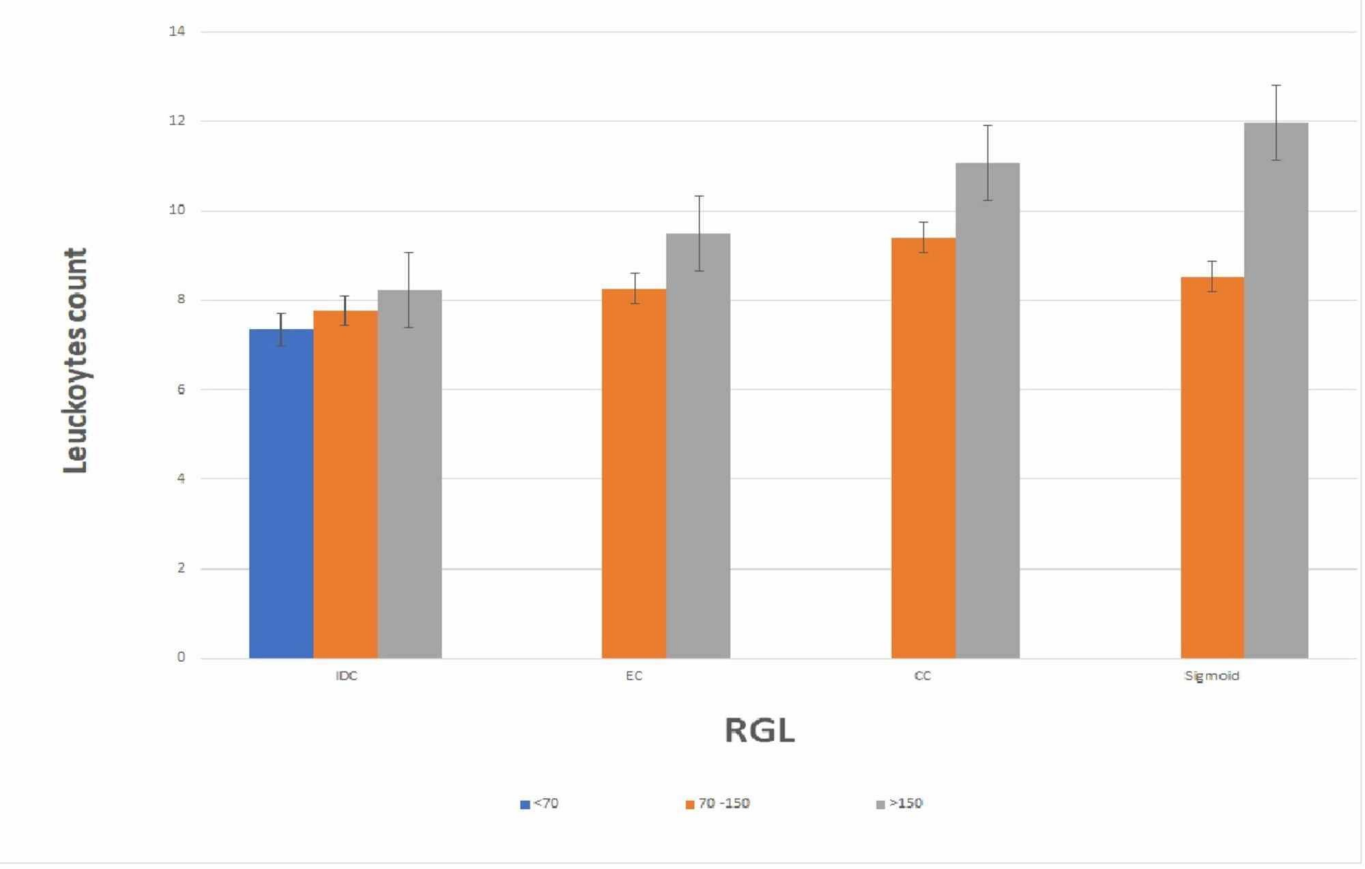
Leukocytes count increases positively with the increase in RGL in blood, highest leukocytes count when >150 RGL (P-value .00001). RGL: Random glucose levels Leukocytes k/uL Number of Invasive duct carcinoma (IDC) <70 = 4, 70 to 150 = 50, >150 = 11 Endometrial carcinoma (EC) 70 to 150 = 26, >150 = 8 Cervical cancer (CC) 70 to 150 = 9, >150 = 3 Sigmoid 70 to 150 = 7, >150 = 3.

In Figure 2, further study about leukocytosis in cancer groups has shown a positive increase in leukocytes count with the increase in RGL in invasive duct carcinoma (IDC), endometrial carcinoma (EC), cervical cancer (CC), and sigmoid cancer (P-value 0.0001). Sigmoid cancer has the highest leukocytosis among the rest of the types.

## Discussion

High glucose levels can increase the proliferation rate of tumor cells. Inflammation is an immune response detected due to infection and tumor development. One of the important features used for diagnoses is inflammation due to tumor-infiltration by leukocytes, where they assist by releasing of several inflammatory mediators. Leukocyte count is a common and affordable test. And it assists in diagnoses, especially in cancer patients. This study has investigated the association between leukocytes count and RGL in female cancer patients. Leukocytes count was higher in those with RGL >150 mg/dL, and the lowest count was detected in those with RGL <70 mg/mL. These findings are consistent with another study, though they have stated that this elevation is an association with a defect in glucose metabolism [14]. Another study demonstrated that cancer mortality is associated with elevation in leukocytes count, where it has reported that this mortality rate is independent of DM, and fasting glucose level [15]. One study found an elevation in leukocytes count before and after the treatment of lung cancer [16]. About 43% of post-menopausal cancer patients have developed leukocytosis, and 30.3% of them are hyperglycemic which was reported by other studies in breast cancer patients and ovarian carcinoma [17,18]. Leukocytosis was higher in patients >40 years old, and especially in post-menopausal females. A study performed by Connolly et al. has detected leukocytosis in several cancer types such as in breast cancer, gastric and pancreatic cancer, lung cancer, and blood cancer [11]. That is consistent with this study findings as leukocytosis was found in 50% of blood tumor patients, 5% of breast cancer patients, where 8% was hyperglycemic, 4% was gastrointestinal tract cancer patients and 11.11% were hyperglycemic. In head and neck cancer patients, leukocytosis was found in 5% of the patients. Squamous cell carcinoma is frequent in head and neck cancer due to factors such as human papillomavirus (HPV) [19]. Many studies have reported leukocytosis in head and neck cancer patients [20]. In female genital tract patients, about 20% had leukocytosis, and 22.22% of hyperglycemic patients had leukocytosis. In female genital tract cancers, leukocytosis is a major prognostic to evaluate survival rates, such as in cervical cancer and endometrial cancer [21-23].

## Conclusions

In conclusion, in this study leukocytosis was positively associated with RGL in female cancer patients. A total of 210 female cancer patients were included in this study with the majority of them showing normal leukocytes count. All hypoglycemic cancer patients did not develop leukocytosis. A significant percentage of hyperglycemic cancer patients developed leukocytosis which is evident that high RGL can lead to an increase in leukocytes count in cancer patients. Leukocytosis was reported to be associated with recurrence of cancer, and this study has detected RGL is also associated with leukocytosis. The recurrence can occur at any time after the treatment. Therefore, the management of glucose levels in these patients is essential.
